# Influence of the Zinc and Fibre Addition in the Diet on Biomechanical Bone Properties in Weaned Piglets

**DOI:** 10.3390/ani12020181

**Published:** 2022-01-12

**Authors:** Grzegorz Skiba, Stanisława Raj, Monika Sobol, Paweł Kowalczyk, Marcin Barszcz, Marcin Taciak, Anna Tuśnio, Klaudia Čobanová, Ľubomira Grešáková, Eugeniusz Ryszard Grela

**Affiliations:** 1 The Kielanowski Institute of Animal Physiology and Nutrition, Department of Animal Nutrition, Polish Academy of Sciences, Instytucka 3, 05-110 Jabłonna, Poland; s.raj@ifzz.pl (S.R.); m.sobol@ifzz.pl (M.S.); p.kowalczyk@ifzz.pl (P.K.); m.barszcz@ifzz.pl (M.B.); m.taciak@ifzz.pl (M.T.); a.tusnio@ifzz.pl (A.T.); 2 Institute of Animal Physiology, Centre of Biosciences of the Slovak Academy of Sciences, Soltesovej 4, 04-001 Košice, Slovakia; boldik@saske.sk (K.Č.); gresakl@saske.sk (Ľ.G.); 3 Institute of Animal Nutrition and Bromatology, University of Life Sciences in Lublin, Akademicka 13, 20-950 Lublin, Poland; eugeniusz.grela@up.lublin.pl

**Keywords:** dietary zinc and fibre, bone parameters, piglets

## Abstract

**Simple Summary:**

The effects of the zinc and fibre source in piglets’ diet on their bone mineral content, density, and strength parameters were investigated using diets supplemented with lignocellulose or potato fibre combined with mineral and organic sources of zinc. The intake of digestible zinc and the zinc content in the blood serum were positively correlated with the measured bone parameters. In conclusion, the bone parameters were affected more strongly by the organic than inorganic source of zinc.

**Abstract:**

The effects of the zinc and fibre source in piglets’ diet on the bone mineral content, density, and strength parameters of the femur were investigated using 24 piglets fed a diet supplemented with either lignocellulose (LC) or potato fibre (PF). Half of each group of piglets consumed a diet with ZnSO_4_ monohydrate or with zinc glycinate (ZnGly). The diets contained similar amounts of lysine, energy, and fibre. Bone mineral content and density were over 9% higher in pigs receiving diets with ZnGly than in animals fed diets with ZnSO_4_. Moreover, ZnGly strongly improved maximum and elastic strength (by 25.7 and 20.0%, respectively, *p* < 0.0001) and bone stiffness (by 29.4%, *p* < 0.0001). Only the mass of the femur was affected by the type of fibre in the diet, as the femurs of piglets fed diets with LC were over 7% (*p* < 0.0001) heavier than in piglets fed diets with PF. The intake of digestible zinc and the zinc content in the blood serum were positively correlated with the measured bone parameters and, depending on the parameter, “r” ranged from 0.749 to 0.866 and from 0.400 to 0.479, respectively. It can be concluded that bone parameters are affected more strongly by the organic than inorganic source of zinc.

## 1. Introduction

Zinc, as an abundant essential trace element, performs multifarious physiological roles and, as a component of many metalloenzymes, is involved in almost every metabolic pathway in the animal [1]. Typically, zinc distribution in the body is about 57% in the muscle, 29% in the bones, 6% in the skin, 5% in the liver, 1.5% in the brain, and 0.1% in the blood plasma [2]. Thus, the skeleton is the second major body store of this element. Zinc is needed to produce the matrix of collagen protein threads upon which the bone-forming calcium–phosphorus compound is deposited. It also acts as a co-factor in over 200 enzymatic reactions involved in carbohydrate, protein, and lipid metabolism [3]. Zinc is required by enzymes playing specific functions in bone metabolism. Alkaline phosphatase (ALP, necessary for bone calcification) and collagenase (essential for bone resorption and remodeling) are zinc metalloenzymes [4]. Zinc also influence the normal physiological action of vitamin D on calcium metabolism and interfere with the anabolic activity of vitamin D on bone tissue [5]. Moreover, zinc stimulates food intake through the afferent vagus nerve followed by activating the hypothalamic peptides (orexin and neuropeptide Y) associated with food intake regulation, according to Ohinata et al. [6]. It is also known that zinc supplementation increases circulating leptin levels, a hormone playing a key role in the regulation of appetite and energy metabolism [7].

Usually, inorganic forms (ZnSO_4_, ZnO, ZnCl_2_, ZnHCl) are the major sources of Zn for animals. However, the bioavailability of such (inorganic) sources of this element has been reported to be quite low [8]. Therefore, recently, great interest has been aroused by sources with higher bioavailability to the animals. Organic forms of zinc exhibit higher bioavailability than inorganic sources, especially in monogastric animals [9]. An important factor for the intestinal absorption of Zn is its availability as free ions in the intestinal lumen [10]. The different forms of Zn are characterised by a large difference in solubility, e.g., zinc sulphate is highly soluble in a wide range of pH from 2.5 to 6.5, whereas zinc chlorate is less soluble at pH 6.5 but highly soluble at pH 2.5 [11]. In turn, zinc oxide and zinc sulphate are more easily dissociated in the acidic pH in the stomach than the chelated form of this element. The dissociation leads to the formation of zinc-phytate complexes [11], which may impair phytase efficacy, resulting in lower phosphorus release from the phytate molecule [12]. Chelated zinc sources are stable in the upper gastrointestinal tract, thus the formation of Zn-phytate complexes is minimised, allowing Zn to be absorbed by epithelial cells in the jejunum and ileum [13], as recently confirmed by Xie et al. [14]. Moreover, the bioavailability of Zn from organic sources depends on the strength of binding between the amino acid and zinc [15].

Potato fibre (PF) is a co-product of potato starch manufacture and is seen as a potential source of functional dietary fibre. Dietary fibre contributes to over 60% of PF dry matter and its main components are cellulose, hemicellulose, and pectins. The inclusion of dietary fibres in feed has become increasingly popular due to their positive effects on modulating intestinal microbiota populations and consequently on the immunology, health, and performance of young rats [16] and pigs [17]. Supplementation of the diet with dietary potato fibre delays the rate of digesta passage without reduction of fat and protein digestibility and improves the digestibility of gross energy, dry matter, and crude fibre, as compared to cellulose and pectin. A recent study conducted by Holodova et al. [18] showed that the dietary intake of potato fibre can influence the intestinal absorption of zinc and other trace elements through an effect on the Zn-transporter levels in the jejunum and increased zinc content in the ileum, which is the main site of non-saturable transport pathways.

As shown recently [19], Zn is an essential mineral required for normal skeletal growth and bone homeostasis. Dietary zinc deficiency has been linked to impaired skeletal development and bone growth [20]. Consistent with reduced skeletal growth, the circulating levels of growth hormone and IGF-1 are reduced during zinc deficiency [21]. Since Zn is located at sites of tissue calcification, including osteons and calcified cartilage, the bone tissue zinc levels increase as bone mineralisation increases [22]. Results of a recent study have shown that dietary supplementation of Zn increased the bone biomechanical properties [23,24]. Contrarily, Świątkiewicz and Koreleski [25] indicated that supplementation of Zn had no effect on the mechanical characteristics of bone. However, the cellular and molecular pathways through which zinc promotes bone growth, homeostasis, and regeneration are poorly understood.

Fibre intake and its impact on bone health are not clear. Some previous studies indicate that the beneficial effects respecting reduced proneness to various degenerative diseases are likely to far outweigh the possible adverse effects of reduced bioavailability of mineral nutrients [26]. Feng et al. [27] suggest that increased consumption of fibre by women leads to hypoestrogenism (an important risk factor for osteoporosis) and can influence bone mass adversely. In turn, results of recent studies have shown that additional intake of 1 g fibre daily increases bone mineral density, however only in young males [28]. Similar results were reported by [29]. However, the authors observed this positive association in different parts of the skeleton.

The growth of the skeleton is closely related to achievement of animal growth potential. Therefore, availability of nutrients (including minerals) is extremely important for young (weaned) pigs. To the best of our knowledge, the interactive effect of fibre and the Zn source on bone mineral parameters in young pigs has never been investigated. The objective of this work was to determine the effect of the Zn source (organic or inorganic) combined with potato fibre (PF) or lignocellulose (LC), on the morphometric (mass), densitometric (bone mineral content and density), and strength parameters of the femur in weaned pigs. Given the role of zinc and dietary fibre in animal nutrition, it was hypothesised that the densitometric, strength and morphometric parameters of the femur of weaned pigs are related to the source of zinc and fibre in their diet.

## 2. Materials and Methods

### 2.1. Ethics

The design of the study, animal care, and all procedures applied during the experiment were performed in accordance with the relevant principles of the European Union and the Polish Animal Protection Act and were approved by the II Local Ethics Committee on Animal Experimentation of Warsaw University of Life Sciences, SGGW, Warsaw, Poland (resolution number WAW2_21/2016). The experiment was designed to minimise the number of animals whilst maintaining high statistical power according to the principles of the 3Rs (replacement, reduction, and refinement).

### 2.2. Animals, Diets, Housing, and Treatment

The research material (femurs) used in the present work was collected from the experiment investigating the effect of organic and inorganic zinc source, used in combination with potato fiber, on growth, nutrient digestibility, and biochemical blood profile in growing pigs [30]. The experiment was carried out on 24 barrows (Danbred × Duroc) at the age of 40 days. Piglets characterized a high growth potential. The study was conducted in the period of the rapid bone growth and mineralization. During this time, animals are very sensitive to experimental factors. Thus, 28 days is enough to induce changes in the degree of bone mineralisation (mineral content and mineral density) and biomechanic properties. Animals were kept individually in pens with free access to feed and water. Through experiment in the piggery the thermoneutral conditions were maintained. The diets were formulated based on cereals (barley 20%, wheat 45%), protein compounds (soybean meal 17%, yellow lupine 6%) and energetic compounds (rapeseed oil 2.55%, corn starch 3.3%) and were supplemented with mineral-vitamin mix, sodium chloride, calcium, and amino acids. All diets were balanced in the content of energy, protein, minerals (including zinc and phosphorus), and amino acids. Lysine content amounted 13.3 g (standardized ileal digestible) per kg dry matter. Remaining essential amino acids (methionine + cysteine: threonine: tryptophan) were balanced in proportion to the lysine content (100:60:70:20). The chemical composition of diets is presented in Table 1.

Experiment was arranged in a 2 × 2 factorial design. Pigs were allocated according to body weight to 4 dietary treatments (6 animals each). For the consecutive 28 days (from 40 to 68 days of age), half of the piglets were fed either a diet supplemented with 1.7% of lignocellulose (Lonocel, Cargill Poland Ltd., Kiszkowo, Poland, group LC) or 5.0% of potato fibre (Potex, Lyckebyh, Kristianstad, Sweden, group PF). In each group, half of the piglets consumed a diet supplemented with a mineral source of zinc (0.033% ZnSO_4_ monohydrate, Sigma-Aldrich Corp., Saint Louis, MO, USA; groups LCM and PFM) and the other animals received an organic source of zinc (0.046% ZnGly, Glycinoplex-Zn 26%, Phytobiotics Futterzusatzstoffe GmbH, Eltville, Germany; groups LCO and PFO). The design of the study is presented in Table 2.

### 2.3. Analysed Parameters

The following data shown in the presented study, namely nutritional value of feed and total and digestible zinc intake, were calculated on the basis of individual performance data of animals obtained by Barszcz et al. [30]. The previously obtained data were also used to calculate the correlation between the zinc intake and the measured bone parameters. The authors also shared individual animal data on the content of zinc and ALP in the blood, which were also used to calculate the relationship between their concentration in the blood and the measured bone parameters.

### 2.4. Femur Collection

At the end of the study, the pigs were slaughtered. Before slaughter, the pigs were electrically stunned (STZ 3 apparatus; P.P.H. MASTER Sp. J., Solec Kujawski, Poland) and then exsanguinated. After exsanguination, the left femur was dissected from the carcass. After excision, the bones were cleaned of any remaining flesh, weighed, and frozen (−20 °C) for subsequent dual-energy X-ray absorptiometry scanning.

### 2.5. Absorptiometric Measurements

Dual-energy X-ray absorptiometric scans of the femur were obtained using the XR-800TM (Norland Medical Systems, CooperSurgical, Fort Atkinson, WI, USA) densitometer scanner according to the manufacturer’s protocol of scanning and analysis (research-scan type). A quality assurance test to verify the stability of the system calibration (control scans) was performed on a daily basis. A daily calibration procedure was performed using a QC Phantom and QA Calibration standard (Norland, A Cooper Surgical Company). Specimens for scanning were thawed at 23 °C for 12 h prior to use. During scanning, the left was positioned horizontally, with the femoral head facing upwards and the condyles facing downwards, and then scanned from the distal to proximal end. All scans were performed in two replicates to avoid bone rotation, as inconsistencies in the orientation can hamper the accuracy of test results. The mean value was used for statistical analysis. To ensure consistency, all scans were performed by the same operator. Bone mineral content (BMC) and bone mineral density (BMD) were recorded.

### 2.6. Three-Point Bending Test

After dual-energy X-ray absorptiometry scanning, the three-point bending test using a TA-HDi Texture Analyser (Stable Micro Systems Ltd., Godalming, UK) was applied to determine the biomechanical properties of the femur. The distance between the supports of the bone was set at 40% of the femur length, and the measuring head loaded the bone samples at the midshaft with a constant speed of 50 mm/min. The values of maximum bone strength and maximum bone elastic strength were determined. The displacement (mm), elastic strength, and maximum strengths (kG) were recorded and femur stiffness was then calculated according to the following formula:

Stiffness (kG/mm) = Elastic strength (kG)/displacement (mm)(1)

### 2.7. Statistical Analyses

Statistical analyses were performed using Statistica (version 13.1, StatSoft, Tulsa, OK, USA). The examined bone characteristics in different groups are presented as mean values. The results were analysed with two-way ANOVA, with the type of fibre and the type of zinc supplement as the main variables. When the F ratio was significant, the Tukey test was used to determine differences between the groups. Statistical significance was set at *p* < 0.05. A borderline significant trend was set at *p* < 0.09. The relationships between digestible zinc and phosphorus intake, alkaline phosphatase, and zinc concentrations in the blood serum vs. bone parameters were analysed using Pearson’s correlation.

## 3. Results

Piglets fed the diet supplemented with zinc glycinate consumed more total Zn (by over 19%, *p* = 0.0163) and tended to consume more digestible Zn (by 6.5%, *p* = 0. 0580) as compared to animals fed the diet with ZnSO_4_ (Table 3). The diets supplemented with PF increased the intake of digestible Zn (by 6%, *p* = 0.0451), in comparison with the LC diets.

The only bone trait affected by the fibre type was the bone mass, as the femora of piglets fed the diet supplemented with lignocellulose were over 7% (*p* = 0.0064) heavier than the femora of piglets fed the potato fibre-supplemented diet (Table 4). In contrast, the source of zinc strongly influenced bone mineral content and bone mineral density, as both parameters were over 9% higher in pigs receiving the diet with zinc glycinate (ZnGly) than in animals fed the diet supplemented with zinc sulphate (ZnSO_4_). Moreover, the supplementation of the diet with the organic form of zinc (ZnGly) strongly improved bone, maximum, and elastic strength (by 25.7 and 20.0%, respectively, *p* = 0.0001), as well as bone stiffness (by 29.4%, *p* = 0.0001).

Positive correlations (Table 5) were detected between digestible zinc intake and bone mineral density (r = 0.866, *p* = 0.0152), bone mineral content (r = 0.749, *p* = 0.0001), maximum bone strength (r = 0.798, *p* = 0.0001), elastic bone strength (r = 0.826, *p* = 0.0001), and stiffness (r = 0.773, *p* = 0.0001). Positive correlations were also found between the concentration of zinc in the blood serum and bone mineral density (r = 0.440, *p* = 0.0560), bone mineral content (r = 0.472, *p* = 0.0528), maximum bone strength (r = 0.400, *p* = 0.0600), and bone stiffness (r = 0.479, *p* = 0.0587). Contrary to expectations, no relationship was found between the concentration of alkaline phosphatase in the blood and the examined biomechanical features of the bones.

## 4. Discussion

Zinc is an essential dietary component for pigs, as it is an important element in the maintenance of the structure of growth hormone and insulin, having a huge impact on animal growth. Moreover, it is an important part of over 200 metalloenzymes [3]. Thus, zinc participates in various molecular mechanisms both at the cellular level and in in vivo systems. Zinc is required for the production of the matrix of collagen protein threads, upon which the bone-forming calcium–phosphorus compound is deposited. This element also stimulates the production of enzymes responsible for the degradation and recycling of worn-out bits of bone protein. Zn may exert an impact on the normal physiological action of vitamin D in calcium metabolism and interfere with the anabolic activity of vitamin D in bone tissue [5].

The results of the present study show that piglets supplemented with zinc glycinate consumed daily more zinc (both total and digestible) as compared to piglets fed the zinc sulphate-supplemented diet. This could result from the increased digestibility of organic zinc, as the research of Xie at al. [14] revealed that partial and complete substitution of inorganic by organic zinc increased the apparent total tract digestibility of zinc.

The results of the present study also demonstrated that the digestible zinc consumption was higher when the diet was supplemented with potato fibre. This response of piglets may probably be explained by the delay in the digesta passage through the alimentary tract [17] probably resulting in more efficient zinc absorption in the small intestine. It is also possible that more efficient fermentation took place in the large intestine and, consequently, the release of native zinc contained in the fibre fractions. However, our experiment was carried out on piglets. Thus, the influence of this phenomenon (process) on total zinc balance is insignificant. Another explanation may be connected with the influence of potato fibre on the solubility of mineral complexes. All these combined factors may result in an increased uptake of digestible zinc. As reported by Holodova et al. [18], the dietary intake of potato fibre can influence the intestinal absorption of zinc and other trace elements due to its effect on the Zn-transporter levels in the jejunum, which is the main site of non-saturable transport pathways. Buff et al. [32] showed that feeding weaned piglets a diet with Zn polysaccharides strongly decreased Zn excretion, compared with a diet containing Zn oxide, which may suggest that zinc polysaccharides can improve zinc absorption. However, a detailed analysis of the results of these authors indicates that this conclusion was due to the fact that piglets received ZnO in a pharmacological dose (several times higher than the groups receiving Zn in the form of a polysaccharide). It seems that it would be more correct to compare the groups receiving Zn-polysaccharide with the control group (with standard Zn content in the form of Zn-SO_4_ without the addition of polysaccharide). Such a comparison shows that the groups receiving Zn-polysaccharide excreted more zinc, with increasing Zn uptake, but were characterized by greater retention and absorption of this element. Unfortunately, the authors of the cited work do not provide the mechanisms explaining such a response of animals. Nevertheless, these data may partially explain the results we obtained (current results).

The results of the present study indicate that the intensity of the positive impact on mineralisation and mechanical parameters of bone depends on the source of dietary zinc. All the biomechanical characteristics of the femur of piglets fed the diet supplemented with zinc glycinate were considerably better than those of the animals fed the diet supplemented with the inorganic source of zinc (ZnSO_4_). An increase in bone mineral content was observed by Li et al. [33] in ovariectomised rats treated with an inorganic source of zinc. Unfortunately, the authors did not use any organic form of zinc. Bortolin et al. [34] showed that supplementation of diabetes rats’ diet with zinc carbonate had a beneficial effect on biomechanical bone parameters compared to diabetes animals not supplemented with such a source of zinc. In turn, Olgun and Yildiz [23] found that the bone characteristics in laying hens and broiler chickens were significantly higher when their diet contained an inorganic compared to organic zinc source. Świąkiewicz and Koreleski [25] found that bone biomechanical properties were not affected by organic zinc supplementation in laying hens. Tüzün et al. [24] found no significant differences in bone strength, stiffness, or mineralisation in quails fed inorganic, organic, and nano zinc sources. Similarly, studies presented by Tomaszewska et al. [22,35] demonstrated that the source of Zn (ZnO vs. ZnGly) in the diet did not affect the femoral mechanical parameter, cross-sectional area, and wall thickness in broilers.

Unfortunately, in the present study, we focused only on the influence of the dietary zinc source on the mineralisation and biomechanical properties of bone, but the mechanism of zinc action was not investigated. However, based on the results of recent studies, it seems that zinc can promote bone growth, bone properties, and homeostasis through a few cellular and molecular pathways. As suggested by Ferreira et al. [36], zinc supplementation reduces the RANKL/OPG ratio and prevents bone architecture alterations. As reported by Liu et al. [37], zinc inhibits osteoclastogenesis and promotes bone formation by stimulating the proliferation and differentiation of osteoblasts. Amin et al., [38] have found that zinc exhibits protective properties against bone loss by suppressing osteoclastogenesis via the downregulation of RANKL/RANK. Additionally, zinc supplementation results in the upregulation of OPG expression. Other authors have found that zinc improves osteoblastic bone mineralisation by activating alkaline phosphatase [20] and stimulating collagen synthesis [39], which improves bone matrix calcification [40]. Moreover, it has also been suggested that Zn supplementation can have a positive impact on bone mineral content and prevent osteoporosis [41], whereas a deficiency of this bio-element increases collagen solubility in the bone tissue, thus reducing the extracellular bone matrix [42]. In turn, as indicated by Suzuki et al. [43], zinc deficiency can down-regulate antioxidants in bone due to iron accumulation, and thus induce oxidative stress and the production of inflammatory cytokines, stimulating the expression of osteoclastogenesis-related genes and, consequently, increased bone resorption. Simultaneously, reduced expression of osteogenic genes (e.g., ALP, Osteocalcin, Collal, Runx2, Osterix) results in decreased bone formation. Based on an in vivo study, Brzóska and Rogalska [44] have evidenced that the beneficial Zn impact on the skeleton can be related to improvement of the bone tissue oxidative/antioxidative status and mediation of the RANK/RANKL/OPG system. Some literature data [35] have indicated that the effect of zinc supplementation on bone development may be related to its direct impact on hormonal growth mediators. These authors reported that the anabolic effect of insulin-like growth factor 1 (IGF-I) on osteoblasts is enhanced by supplementation of a diet with organic zinc. They suggested that the higher bioavailability and absorption of ZnGly not only causes greater deposition of zinc in the bone tissue, but also influences cell metabolism/function through its effects on hormones and growth factors. These authors also suggest that IGF-1 locally produced in tissues plays a more important role in bone metabolism than the circulating IGF-1 produced by the liver. Recent literature data have also indicated that the serum zinc level and dietary zinc intake may play an essential role in preventing osteoporosis via the anabolic effect on osteoblast formation in the marrow [33] or increased activity of protein molecules considered as markers of bone formation [45].

The present data showed that the dietary fibre source (lignocellulose or potato fibre) had an effect only on the femur mass in the piglets, whereas the other parameters of bones did not differ between the groups. Fibre intake and its clinical impact on bone health have been investigated mainly in humans, but the results and mechanisms of action are not clear-cut. Some previous experimental studies indicate that the beneficial effects respecting reduced proneness to various degenerative diseases are likely to far outweigh the possible adverse effects of reduced bioavailability of mineral nutrients [26]. Moreover, Feng et al. [27] suggest that increased consumption of fibre by women leads to hypoestrogenism (an important risk factor for osteoporosis) and can influence bone mass adversely. In turn, results of recent studies [28] have demonstrated that additional intake of 1 g fibre daily increases bone mineral density by 0.004 g/cm^2^, however only in young males. Similar results were reported by Dai et al. [29]. However, authors observed this positive association in different parts of the skeleton. The positive impact of dietary fibre on bones may be explained by its positive effect on the gut environment (e.g., increased intestinal lumen, height of intestinal villi, and depth of crypts) [46] and by the production of short-chain fatty acids, which are a product of its fermentation [47]. Both factors have a positive influence on calcium absorption.

Data concerning the influence of zinc source on zinc concentration in the blood are ambiguous. Based on the results of studies with ruminants [48], poultry [49], guinea pigs [50], and piglets [30,32], it can be stated that dietary Zn source does not affect blood zinc concentration. The results reported by Garg et al. [51] indicated that supplementation of Zn from organic or inorganic sources in a diet containing low zinc significantly increased the serum Zn concentration in lambs supplemented with organic zinc. Similar findings were presented by Yan et al. [52] in a study with rabbits. The results of the cited studies indicate that organic zinc supplemented to the diet can generally increase the concentration of this element in blood serum. However, other factors, such as the type of the organic form and its bioavailability and the level/amount of supplementation, may also play an important role. In the present study, the analysis of the relationship between digestible zinc intake, bone mineralisation parameters (mineral content and mineral density), and bone strength parameters (both maximum and elastic strength and stiffness) showed a strong positive correlation. The concentration of zinc in bone is higher than that in most tissues, thus it is not surprising that an earlier study, although carried out in humans, also found a positive correlation between the degree of bone mineralisation and zinc intake [53]. Other authors [54] found that zinc intake in postmenopausal women was positively associated with bone mineral density and that the values of the T-score of the lumbar spine, femoral neck, and the total hip were positively correlated with the intake of zinc. Similar results of an earlier study with osteoporotic men [55] and women [53] showed that both the dietary zinc intake and the plasma zinc concentration were correlated positively with bone mineral density, as the dietary zinc intake and plasma zinc concentrations were lower in individuals with osteoporosis than in healthy individuals. This association was cross-sectional, longitudinal, and independent of the age or body mass index.

Alkaline phosphatase is a zinc dependent enzyme recognised as a marker of bone formation. Literature data indicated that Zn deficiency significantly decreased ALP activity in the plasma of rats [56,57] and pigs [58], which in turn could indicate a decreased bone formation process. Data revealed by Prins et al. [59] showed the organic zinc (Zn glycinate) enhancement of the osteoblast proliferation and bone forming capacity of bone marrow, as indicated by the increased concentration of ALP. Consequently, the retention of minerals in the bone could be improved. Surprisingly, in the present results, the correlation coefficients between the zinc and ALP content in the blood serum and properties of femur (mineralization and biomechanic) were found to be insignificant.

## 5. Conclusions

It can be concluded that the supplementation of a diet with an organic source of zinc positively influences the degree of bone mineralisation and bone strength parameters. This impact is greater than that of inorganic sources of this element. Moreover, the degree of bone mineralisation and mechanical properties are more strongly correlated with the digestible zinc intake than to the zinc and alkaline phosphatase concentrations in blood serum.

## Figures and Tables

**Table 1:** Composition and energetic value of the diets.

Item	LCM	LCO	PFM	PFO
Chemical composition, g/kg dry matter				
Crude ash	53	52	55	55
Crude protein	205	206	207	205
Fat (ether extract)	48	46	47	45
Crude fibre	47	45	45	45
Total phosphorus	5.7	5.8	5.7	5.7
Zinc	0.160	0.160	0.158	0.160
Energetic value, MJ/kg dry matter				
Gross energy,	18.8	18.9	18.8	18.6
Metabolisable energy *	14.6	14.7	14.6	14.4

LCM—lignocellulose diet supplemented with ZnSO_4_; LCO—lignocellulose diet supplemented with ZnGly; PFM—potato fibre diet supplemented with ZnSO_4_; PFO—potato fibre diet supplemented with ZnGly; * Metabolisable energy was calculated according to Kirchgessner and Roth [31].

**Table 2:** Experimental design.

Zinc Source	Fibre Source	
	Lignocellulose (LC)	Potato Fibre (PF)
0.033% ZnSO_4_ (Inorganic)	LCM (*n* = 6)	PFM (*n* = 6)
0.046% ZnGly (Organic)	LCO (*n* = 6)	PFO (*n* = 6)

LCM—lignocellulose diet supplemented with ZnSO_4_; LCO—lignocellulose diet supplemented with ZnGly; PFM—potato fibre diet supplemented with ZnSO_4_; PFO—potato fibre diet supplemented with ZnGly.

**Table 3:** Daily intake of total and digestible zinc by piglets during experiment.

Parameters	Type of Fibre	Type of Zinc		Mean	SEM	*p*-Value		Interaction
		Organic ZnGly	Mineral ZnSO_4_			Type of		
						Fibre	Zinc	
Total zinc, mg	PF	142	112	127	3.28	0.4552	0.0163	ns
	LC	142	126	134				
	Mean	142	119	131				
Digestible Zn, mg	PF	45.4	40.1	42.8	0.825	0.0451	0.0580	ns
	LC	39.3	39.6	39.5				
	mean	42.4	39.8	41.1				

PF—diet supplemented with potato fibre; LC—diet supplemented with lignocellulose; SEM—standard error of the mean.

**Table 4:** Femur mass, mineral density, mineral content, maximum and elastic strength, and stiffness measured at the end of the study.

Parameters	Type of Fibre	Type of Zinc		Mean	SEM	*p*-Value		Interaction
		Organic ZnGly	Mineral ZnSO_4_			Type of		
						Fibre	Zinc	
Mass, g	PF	121.88	112.33	117.11	1.970	0.0064	0.3022	ns
	LC	123.89	127.54	125.72				
	Mean	122.89	119.94	121.41				
Bone mineral density, g/cm^2^	PF	0.550	0.483	0.517	0.009	0.1982	0.0030	ns
	LC	0.509	0.488	0.499				
	Mean	0.530	0.485	0.508				
Bone mineral content, g	PF	21.97	18.75	20.36	0.444	0.9050	0.0132	ns
	LC	20.59	20.24	20.42				
	Mean	21.28	19.50	20.39				
Maximum strength, kG	PF	158.9	103.4	131.2	7.13	0.5790	0.0001	ns
	LC	129.7	126.2	128.0				
	Mean	144.3	114.8	129.6				
Elastic strength, kG	PF	113.6	80.1	96.9	4.31	0.3913	0.0001	ns
	LC	95.4	94.1	94.8				
	Mean	104.5	87.1	95.9				
Stiffness, kG/mm	PF	56.8	37.3	47.1	2.38	0.5391	0.0001	ns
	LC	47.7	43.4	45.6				
	Mean	52.3	40.4	46.3				

PF—diet supplemented with potato fibre; LC—diet supplemented with lignocellulose; SEM—standard error of the mean.

**Table 5:** Pearson’s correlation coefficient between bone parameters vs. the daily intake of Zinc and the content of zinc and alkaline phosphatase in the blood serum.

Item	Intake of Digestible Zn	Concentration in the Blood Serum	
		Zinc	Alkaline Phosphatase
Bone mineral density	0.866 (*p* = 0.0152)	0.440 (*p* = 0.0560)	0.549 (*p* = 0.4514)
Bone mineral content	0.749 (*p* = 0.0001)	0.472 (*p* = 0.0528)	0.777 (*p* = 0.2231)
Maximum bone strength	0.798 (*p* = 0.0001)	0.400 (*p* = 0.0600)	0.745 (*p* = 0.2563)
Elastic bone strength	0.826 (*p* = 0.0001)	0.377 (*p* = 0.6230)	0.744 (*p* = 0.2650)
Bone stiffness	0.773 (*p* = 0.0001)	0.479 (*p* = 0.0587)	0.694 (*p* = 0.3050)

## Data Availability

The data supporting reported results are in the possession of the Authors (G.S., S.R. and M.B.).
